# The predicting role of circulating tumor DNA landscape in gastric cancer patients treated with immune checkpoint inhibitors

**DOI:** 10.1186/s12943-020-01274-7

**Published:** 2020-10-30

**Authors:** Ying Jin, Dong-Liang Chen, Feng Wang, Chao-pin Yang, Xu-Xian Chen, Jin-qi You, Jin-Sheng Huang, Yang Shao, Dong-Qin Zhu, Yu-Ming Ouyang, Hui-Yan Luo, Zhi-Qiang Wang, Feng-Hua Wang, Yu-Hong Li, Rui-Hua Xu, Dong-Sheng Zhang

**Affiliations:** 1 State Key Laboratory of Oncology in South China, Collaborative Innovation Center for Cancer Medicine, Sun Yat-sen University Cancer Center, Sun Yat-sen University, Guangzhou, 510060 P. R. China; 2 Department of Medical Oncology, Sun Yat-sen University Cancer Center, Guangzhou, 510060 Guangdong China; 3 Department of Biotherapy, Sun Yat-sen University Cancer Center, Guangzhou, 510060 Guangdong China; 4 Department of VIP region, Sun Yat-sen University Cancer Center, Guangzhou, 510060 Guangdong China; 5 Medical Department, Nanjing Geneseeq Technology Inc., Nanjing, 210032 Zhejiang China; 6 School of Public Health, Nanjing Medical University, Nanjing, 211166 Zhejiang China

**Keywords:** Circulating tumor DNA, Immune checkpoint inhibitors, Gastrointestinal cancer, Advanced, Biomarkers

## Abstract

A more common and noninvasive predicting biomarker for programmed cell death 1 (PD-1) antibody remains to be explored. We assessed 46 patients with advanced gastric cancer who received PD-1 antibody immunotherapy and 425-genes next-generation sequencing (NGS) testing. Patients who had a > 25% decline in maximal somatic variant allelic frequency (maxVAF) had a longer progression free survival (PFS) and higher response rate than those who did not (7.3 months vs 3.6 months, *p* = 0.0011; 53.3% vs 13.3%, *p* = 0.06). The median PFS of patients with undetectable and detectable post-treatment circulating tumor DNA (ctDNA) was 7.4 months vs. 4.9 months (*p* = 0.025). Mutation status of TGFBR2, RHOA, and PREX2 in baseline ctDNA influenced the PFS of immunotherapy (*p* < 0.05). Patients with alterations in CEBPA, FGFR4, MET or KMT2B (*p* = 0.09) gene had greater likelihood of immune-related adverse events (irAEs). ctDNA can serve as a potential biomarker of the response to immunotherapy in advanced gastric cancers, and its potential role in predicting irAEs worth further exploration.

**Supplementary Information:**

**Supplementary information** accompanies this paper at 10.1186/s12943-020-01274-7.

Gastric cancer is the fifth most common malignant tumor and the third leading cause of cancer-related death worldwide [1]. The immune Checkpoint Inhibitors (ICIs), mainly the antibodies against PD-1, have been recommended as a palliative therapy option for selective patients with metastatic gastric adenocarcinoma, with predictive biomarkers including the microsatellite instability (MSI) status, Combined Positive Score (CPS) of PD-L1 expression, and Epstein-Barr virus (EBV) status of tumor [2, 3]. Our team have identified tumor mutation burden (TMB) as a biomarker for OS benefit in chemo-refractory gastric cancer treated with PD-1 antibody [4]. However, in real-world practice, patients with advanced gastric cancer may lack fresh tissue and have to obtained tissue samples from invasive re-biopsy.

ctDNA has been reported to have utility in identifying genetic alterations and predicting the prognosis, identifying resistance of target therapy, and monitoring relapse or progression of gastric cancer [5]. What’s more, ctDNA could provide longitudinal and dynamic surveillance of the tumor-specific genetic characteristics without repeatedly performing invasive tumor biopsy that costs more time and money. Recently, the use of dynamic ctDNA to predict the response of melanoma and non-small cell lung cancer (NSCLC) to checkpoint inhibitors has been reported [6, 7], while in gastric cancer little has been explored. In this study, we aim to explore the predicting role of ctDNA in gastric cancer patients treated with immunotherapy.

## Results and discussion

### Clinicopathologic characteristics and genomic landscape of ctDNA-NGS

Totally, forty-six eligible patients with metastatic gastric cancer were enrolled between October of 2018 and December of 2019(Additional file 1: Supplementary materials and methods). Baseline clinicopathologic characteristics are summarized in Additional file 2: Table S1 and the procedure of data analysis was described in Additional file 3: Fig. S1; It comprised of 30 males and 16 females. The majority (78.3%) of patients had metastatic sites of 1 or 2, and common metastatic site was peritoneum (71.7%). In 26 patients with available PD-L1 status, 13 patients had tumors with PD-L1 CPS ≥ 1, and 7 with CPS ≥ 10; 25 patients had an EBV in situ hybridization test, all of which were negative. One patient was MSI-H, and 4 patients were HER-2 positive. More than half (58.7%) of the patients received chemotherapy and immunotherapy as first-line palliative therapy, while others received the treatment as second-line or late line therapy. The chemotherapy included fluorouracil, capecitabine, S-1, albumin-bound paclitaxel, irinotecan, and trastuzumab. The PD-1 antibody included Nivolumab, Pembrolizumab, Toripalimab, and Sintilimab.

**Fig. 1:**
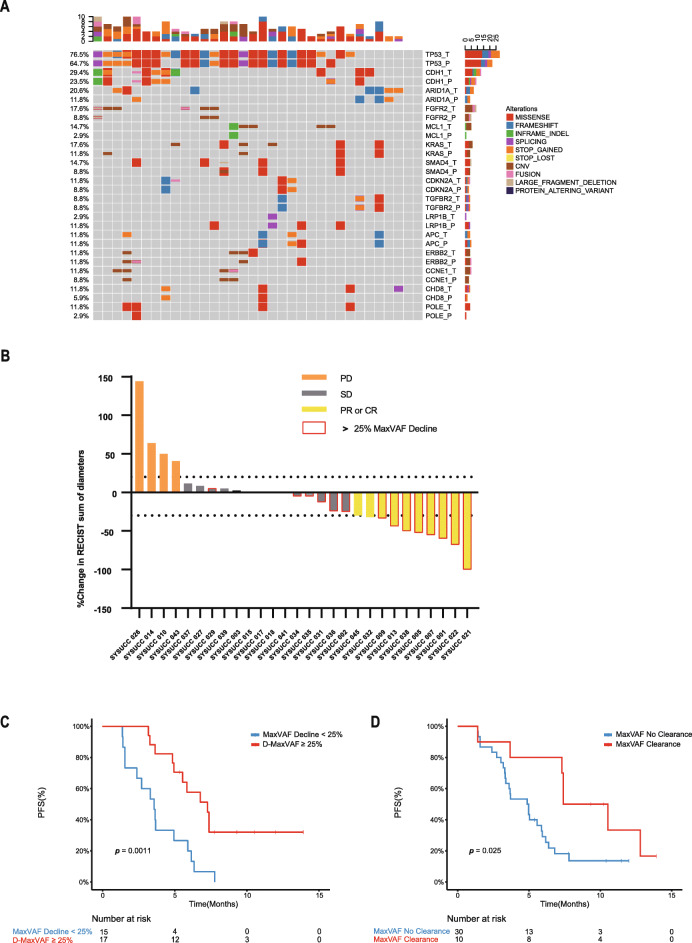
Changes of ctDNA could predict the response to immunotherapy. **a** The landscape of high-frequency genomic alterations detected in 34 paired tissue and baseline plasma samples. **b** Patients who had a > 25% decline in maxVAF had a significant longer PFS than those that did not. **c** Patients with undetectable ctDNA of post-treatment plasma samples had a better PFS. **d** Waterfall plot of best radiologic response and ctDNA response (Only changes of measurable tumors were displayed in this figure). maxVAF, maximal somatic variant allelic frequency

Plasma circulating tumor DNA sequencing (ctDNA-NGS) and tissue tumor DNA sequencing (tissue-NGS) results were obtained using the commercially available 425-gene NGS panel (Fig. 1, Additional file 4: Fig. S2). Of all 46 patients, 38 had tissue samples and 43 had baseline blood samples (Additional file 5: Fig. S3). Among these 43 patients evaluated for baseline ctDNA, 88.4% had at least one genomic alteration (Additional file 5: Fig. S3, Additional file 6: Fig. S4) and 11.6% had undetectable baseline ctDNA. The consistency of ctDNA-NGS with tissue-NGS were also explored (Additional file 7: Fig. S5).

### Decreasing ctDNA was correlated to higher response to immunotherapy

We further analyzed the ctDNA data of patients who had detectable baseline ctDNA and underwent serial ctDNA-NGS (*N* = 32) and found that patients who had a > 25% decline in maxVAF (*N* = 17) had a significantly longer PFS than those who did not (7.3 months; 95%CI, 2.4–4.8 months vs 3.6 months; 95%CI 4.6–10.0 months; *p* = 0.0011; time between serial blood samples collection: median 68 days; rang 19–252 days; Fig. 1). The ORR of patients who had a > 25% decline in maxVAF was 53.3%, higher than those who did not (13.3%; *p* = 0.06). These findings were in consistency with the results of a post hoc exploratory analysis of a prospective phase 2 clinical trial, the change in ctDNA post-treatment were reported to predict response to pembrolizumab, and 14 gastric cancer patients with decreasing ctDNA demonstrated significant improvements in DCR, ORR, and PFS [2]. We identified 10 patients with undetectable and 30 patients with detectable ctDNA of post-treatment plasma samples. The median PFS of patients with undetectable and detectable post-treatment ctDNA was 7.4 months (95%CI, 4.7–10.1 months) vs. 4.9 months (95%CI, 3.1–6.6 months; *p* = 0.025; Fig. 1), respectively.

### Molecular landscape of ctDNA predicts resistance to immunotherapy

**Fig. 2:**
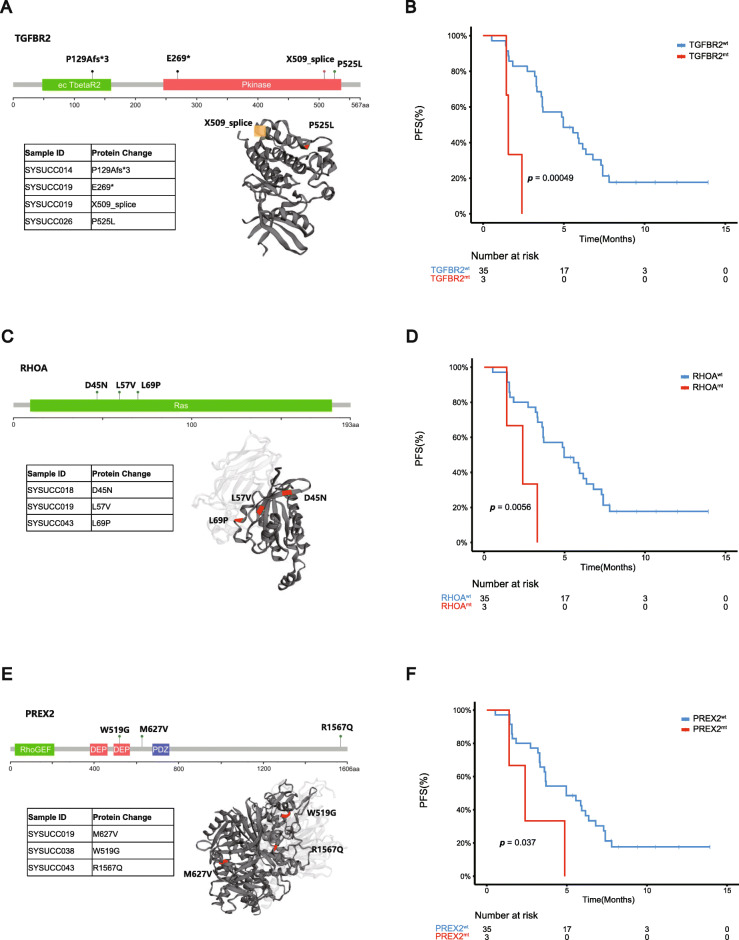
Mutation status of TGFBR2, RHOA, and PREX2 in baseline ctDNA influenced the PFS of immunotherapy. **a** Summary of TGFBR2 mutations (P129Afs*3 and E269* were truncating mutations which could not displayed in the three-dimensional structure of the protein). **b** PFS analysis for TGFBR2 mutations in baseline plasma ctDNA. **c** Summary of RHOA mutations. **d** PFS analysis for RHOA mutations in baseline plasma ctDNA. **e** Summary of PREX2 mutations. **f** PFS analysis for PREX2 mutations in baseline plasma ctDNA

Response evaluations were available for all patients with a median follow-up of 10.4 months. One (2.1%) patient achieved complete response (CR) with the pathological result of the resected tumor indicating a pathological CR (pCR), ten (21.7%) achieved partial response (PR), twenty-twelve (47.8%) had stable disease (SD), six (13.0%) were nonCR/nonPD, and seven (15.2%) patients were progressive disease (PD). In 26 patients with available PD-L1 status, the median PFS of those with PDL1 CPS ≥ 10 and CPS < 10 were 3.4 and 4.9 months (*p* > 0.05). In the patients received first-line treatment, the median PFS of those with PDL1 CPS ≥ 10 and CPS < 10 were 7.8 and 7.4 months(*p* > 0.05). In the second-line or late line setting, there’s also no significant difference between the median PFS in patients with different CPS, which may be due to the small number of patients in each subgroup. Of 43 baseline ctDNA-NGS, we excluded 5 cases with undetectable ctDNA to further explore the correlation of baseline ctDNA-NGS with the duration of immunotherapy, and found the mutation status of TGFBR2, RHOA, and PREX2 influenced the PFS of immunotherapy (Fig. 2). TGFBR2 p.P525L, p.P129Afs*3, p.E269* mutations had been identified and the median PFS of patients with TGFBR2^wt^ and TGFBR2^mt^ were 5.0 months (95%CI, 2.5–7.4 months) and 1.6 months (95%CI, 1.4–1.8 months; *p* < 0.001), respectively. The genomic alteration of the TGFBR2 gene that discovered in our study as a candidate biomarker of immunotherapy had been reported to be related to the responses of anti-pd-L1 treatment (atezolizumab) in patients with metastatic urothelial cancer [8]. RHOA p.L57V, p.L69P, p.D45N mutations had been identified and the median PFS of patients with RHOA^wt^ and RHOA^mt^ were 4.9 months (95%CI, 2.5–7.4 months) and 2.4 months (95%CI, 0.8–4.0 months; *p* = 0.0056), respectively. PREX2 p.M627V, p.W519G, p.R1567Q mutations had been identified and the median PFS of patients with PREX2^wt^ and PREX2^mt^ were 5.0 months (95%CI,2.0–8.0 months) and 2.4 months (95%CI, 0.5–4.3 months; *p* = 0.037), respectively. The PREX2 mutations were reported to enhance cell proliferation in melanoma, hepatocellular carcinoma and pancreatic ductal adenocarcinoma, while its role in immunotherapy had not been explored. Patients with PREX2 mutation tended to have lower PD-L1 CPS and fewer CD8+ T cells (Although not statistically significant, it might due to the limited PREX2 mutant samples), which indicated PREX2 mutation might have potential influence on tumor immune microenvironment and this is worthy of further exploring (Additional file 8: Fig. S6).

To explore the acquired resistance of immunotherapy, we collected plasma samples at PD of the patients who had benefit from the treatment (achieved CR, PR, or SD) and the plasma samples of 16 patients were available. Most of the ctDNA-NGS at PD were largely increased compared with baseline or ctDNA from plasma collected last time (Additional file 9: Table S2), and new genomic alterations were observed in 13 patients. We further compared the newly emerged genomic alterations in ctDNA-NGS with their baseline tissue DNA-NGS. Of the 13 patients with new alterations in plasma, excluding 3 patients that did not have tissue samples, 7 patients had new genomic alterations not found in baseline tissue, and 3 patients had new alterations both could not be found and could be found in baseline tissue. Mutation of FOXL2 gene and CNV of FGFR2 were identified as new alterations in two patients, and RHOA mutations that were discussed above was also found in one patient with acquired resistance, suggesting that these alterations were candidate genes involving in the acquired resistance to immunotherapy.

### Immune-related adverse events of immunotherapy

In this study, most common irAEs were endocrine (10.9%) and hepatic toxicities (8.7%). Severe irAEs that led to termination of immunotherapy were occurred in 2 (4.3%) patients. Some literatures suggested onset of irAE was a biomarker for immunotherapy response, and patients experienced irAEs showed improvements in PFS, OS and ORR [9]. However, in our studies, no marked difference of PFS or ORR were observed between patients with and without irAEs. We investigated the correlations between ctDNA and irAEs, and found that patients with alterations in CEBPA (100% vs 27.3%, *p* = 0.09), FGFR4(100% vs 27.3%, *p* = 0.09), MET (100% vs 27.3%, *p* = 0.09), or KMT2B(100% vs 27.3%, *p* = 0.09) in baseline plasma had greater likelihood of experiencing irAEs, which requires further independent validation in a clinical trial setting with more patients enrolled. Previous studies showed that plentiful biomarkers, including gender, serum cytokines, immune cells, and autoantibodies, were associated with the occurrence of high-grade irAEs [10]. However, there is not any biomarker with high grade of evidence level, more predictive biomarkers of irAEs deserved to be investigated.

## Conclusions

In conclusion, the result of our study indicated that dynamic ctDNA can serve as a potential biomarker of the response to immunotherapy in advanced gastric cancers, and its potential role in detecting the resistance mechanisms and predicting irAEs worth further exploration.

## Supplementary Information

**Additional file 1.** Supplementary materials and methods.

**Additional file 2: Table S1.** Baseline clinicopathologic characteristics.

**Additional file 3: Figure S1.** CONSORT diagram. CONSORT diagram of 46 patients enrolled and samples analyzed.

**Additional file 4: Figure S2.** 425-gene NGS panel that used for plasma circulating tumor DNA sequencing and tissue tumor DNA sequencing.

**Additional file 5: Figure S3.** The landscape of high-frequency genomic alterations detected in 38 tissue samples and 38 baseline plasma samples. High-frequency means genomic alterations detected in tissue or baseline plasma > 5%.

**Additional file 6: Figure S4.** The characteristics of 38 patients with available baseline plasma samples.

**Additional file 7: Figure S5.** Scatter plot between tTMB and bTMB. The Spearman’s rank test showed significant correlations. (*p* < 0.05) **a** Scatter plot between tTMB and bTMB in 24 patients who received immunotherapy as first-line treatment. **b** Scatter plot between tTMB and bTMB in all 38 patients whose tissue and baseline plasma samples were available.

**Additional file 8: Figure S6.** Pathological examination of the patient’s gastric carcinoma in PREX2 wild type and mutant patients. **a**,**d** Representative section of H.E. staining showing gastric carcinoma in wild type and mutant patients respectively. Scale bar: 100 μm. **b**,**e** The PREX2 expression in tumor and non-tumor regions in wild type and mutant patients respectively. Scale bar: 100 μm. **c**,**f** CD8+ T cell densities in wild type and mutant patients respectively. Scale bar: 100 μm. **g** Statistical analysis of PD-L1 expression in 25 MSS patients by combined positive score (CPS). **h** Comparisons of the number of CD8+ T cells in PREX2 wild type and mutant patients. Student’s t-test were used. *p* > 0.05 was not considered statistically significant.

**Additional file 9: Table S2.** The dynamic changes of ctDNA of patients had benefit from the treatment.

## Data Availability

All the data that support the findings of this study are available from the corresponding authors upon reasonable request.

## Abbreviations

PD-1: Programmed cell death 1
NGS: Next-generation sequencing
maxVAF: Maximal somatic variant allelic frequency
ctDNA: Circulating tumor DNA
PFS: Progression free survival
irAEs: Immune-related adverse events
ICIs: Immune Checkpoint Inhibitors
MSI: Microsatellite instability
CPS: Combined Positive Score
EBV: Epstein-Barr virus
TMB: Tumor mutation burden
EGJ: Esophagogastric junction
