# Supplementary material for: The predicting role of circulating tumor DNA landscape in gastric cancer patients treated with immune checkpoint inhibitors
Source: Mol Cancer. 2020 Oct 30;19:154. doi: 10.1186/s12943-020-01274-7 (PMC7596978; doi:10.1186/s12943-020-01274-7)
Supplement: Supplementary file 1 — Additional file 1. Supplementary materials and methods. [file 12943_2020_1274_MOESM1_ESM.docx]

**Supplementary Materials and Methods**

**Patients and study procedure**

We included patients with metastatic gastric cancer from the Sun Yat-Sen University Cancer Center, who started anti-PD-1 immunotherapy with or without systematic chemotherapy between Oct 2018 and Dec 2019 and whose tumor tissue or plasma was available for NGS. Inclusion criteria were as following: (a) diagnosed as metastatic gastric or esophagogastric junction (EGJ) adenocarcinoma; (b) aged over 18 years old; (c) naive to any immunotherapy; (d)adequate organ function to receive treatment. Pre-treatment evaluation included computed tomography (CT) scan or Magnetic Resonance Imaging (MRI), 12-lead ECG, and laboratory tests. Evaluation of the tumor by CT or MRI was performed every 6-8 weeks from first dose until disease progression. This study was approved by the Institutional Review Board of Sun Yat-Sen University Cancer Center, and patients provided written informed consent. The study was compliant with the ethical standards of the institutional and/or national research committee and with the Declaration of Helsinki.

**Tumor and blood sample collection**

Formalin-fixed, paraffin-embedded (FFPE) tumor samples (punctured tissue or surgical specimen) were collected from the 38 of 46 patients. Only FFPE samples that harbored tumor cell content above 20% were considered qualified and sent to the core facility of Nanjing Shihe Jiyin Biotechnology Inc. (Nanjing, China) for further analysis. Totally 110 blood samples for ctDNA were obtained at baseline, when patients achieved a PR or SD, or after disease progression. Each time, about 5 to 10 mL of peripheral blood was collected from each patient in EDTA-coated tubes (BD Biosciences). Plasma was extracted within 2 hours of blood collection and shipped to the central testing laboratory.

**DNA extraction**

About 5-8 of 10μm tissue sections from tumor samples were used for genomic DNA extraction with QIAamp DNA FFPE Tissue Kit (QIAGEN) following the manufacturer’s instructions. Genomic DNA of cellular sediments of pleural effusions were prepared with DNeasy Blood & Tissue kit (QIAGEN). Normal tissue DNA was sequenced together with tumor DNA samples for the purpose of identifying germline mutations. The DNA quality was assessed by Nanodrop2000 (Thermo Fisher Scientific) and the quantity was measured by dsDNA HS Assay Kit (Life Technologies) on Qubit 2.0.

**Targeted NGS and data processing**

Genomic DNA from FFPE sections or biopsy samples and the whole blood control samples were extracted with QIAamp DNA FFPE Tissue kit and DNeasy Blood and tissue kit (Qiagen, USA), respectively. Circulating cell-free DNA (cfDNA) from plasma was extracted using the QIAamp Circulating Nucleic Acid kit (Qiagen). Sequencing libraries were prepared using the KAPA Hyper Prep Kit (KAPA Biosystems) according to manufacturer's instructions for different sample types. customized xGen lockdown probes (Integrated DNA Technologies) targeting 425 cancer-relevant genes were used for hybridization enrichment. The capture reaction was performed with Dynabeads M-270 (Life Technologies) and xGen Lockdown hybridization and wash kit (Integrated DNA Technologies) according to manufacturers’ protocols. Libraries were quantified by qPCR using KAPA Library Quantification kit (KAPA Biosystems). Library fragment size was determined by Bioanalyzer 2100 (Agilent Technologies). The target-enriched library was then sequenced on the HiSeq4000 NGS platform (Illumina) according to the manufacturer’s instructions. The mean coverage depth was 143X for the whole blood control samples, and 1200X for tumor tissues. For cfDNA samples, the mean coverage sequencing depth was 4000X.

**Sequence alignment and processing**

Base calling was performed on bcl2fastq v2.16.0.10 (Illumina, Inc.) to generate sequence reads in FASTQ format (Illumina 1.8+ encoding). Quality control (QC) was performed with Trimmomatic. High quality reads were mapped to the human genome (hg19, GRCh37 Genome Reference Consortium Human Reference 37) using the BWA aligner 0.7.12 with BWA-MEM algorithm and default parameters to create SAM files. Picard 1.119 was used to convert SAM files to compressed BAM files which were then sorted according to chromosome coordinates. The Genome Analysis Toolkit (GATK, version 3.4-0) was used to locally realign the BAMs files at intervals with indel mismatches and recalibrate base quality scores of reads in BAM files.

**SNVs / Indels / CNVs detections**

Single nucleotide variants (SNVs) and short insertions/deletions (indels) were identified by VarScan2 2.3.9 with minimum variant allele frequency threshold set at 0.01, and p-value threshold for calling variants set at 0.05 to generate Variant Call Format (VCF) files. All SNVs/indels were annotated with ANNOVAR, and each SNV/indel was manually checked on the Integrative Genomics Viewer (IGV). Copy number variations (CNVs) were detected using in-house-developed software.

**Statistical analysis**

Comparisons of proportion between groups were done using the Fisher's exact test. Correlations between the tissue TMB (tTMB) and the blood TMB (bTMB) were calculated using the Spearman's rank test. For survival analyses, Kaplan–Meier curves were compared using the log-rank test, and hazard ratios (HR) were calculated by Cox proportional hazards model. Reverse Kaplan Meier method were used to calculated the median follow-up time. A two-sided P value of less than 0.05 was considered significant for all tests unless indicated otherwise. All statistical analyses were done in R (v.3.3.2). The procedure of data analysis was described in Fig S1.
