# Supplementary material for: The predicting role of circulating tumor DNA landscape in gastric cancer patients treated with immune checkpoint inhibitors
Source: Mol Cancer. 2020 Oct 30;19:154. doi: 10.1186/s12943-020-01274-7 (PMC7596978; doi:10.1186/s12943-020-01274-7)
Supplement: Supplementary file 2 — Additional file 2: Table S1. Baseline clinicopathologic characteristics. [file 12943_2020_1274_MOESM2_ESM.docx]

**Table S1. Baseline clinicopathologic characteristics.**

| Character | Number |  |
| --- | --- | --- |
| Number of patients | 46 |  |
| Median age, years (range) | 52 (26-75) |  |
| Gender |  |  |
| Male | 30 | 65.2% |
| Female | 16 | 34.8% |
| Race |  |  |
| Asian | 46 | 100.0% |
| ECOG performance status |  |  |
| 0 | 25 | 54.3% |
| 1 | 21 | 45.7% |
| Family history |  |  |
| Gastric cancer | 11 | 23.9% |
| Other cancers | 11 | 23.9% |
| No | 24 | 52.2% |
| Primary tumor site |  |  |
| EGJ/Cardia | 9 | 19.6% |
| Body | 15 | 32.6% |
| Antrum | 19 | 41.3% |
| Unknown | 3 | 6.5% |
| Histological grade |  |  |
| Moderately differentiated | 14 | 30.4% |
| Poorly differentiated | 31 | 67.4% |
| Unknown | 1 | 2.2% |
| Lauren Classification |  |  |
| Intestinal | 6 | 13.0% |
| Diffuse | 20 | 43.5% |
| Mixed | 8 | 17.4% |
| Unknown | 12 | 26.1% |
| Metastatic sites |  |  |
| Peritoneum | 33 | 71.7% |
| Lymph nodes | 25 | 54.3% |
| liver | 13 | 28.3% |
| Bone | 6 | 13.0% |
| Others | 7 | 15.2% |
| Number of metastatic sites |  |  |
| 1 | 19 | 41.3% |
| 2 | 17 | 37.0% |
| 3 or more | 10 | 21.7% |
| Treatment |  |  |
| First-line | 27 | 58.7% |
| Second-line | 14 | 30.4% |
| third-line or subsequent | 5 | 10.9% |
| Previous gastrectomy |  |  |
| Yes | 13 | 28.3% |
| No | 33 | 71.7% |
| HER-2 status |  |  |
| Positive | 4 | 8.7% |
| Negative | 42 | 91.3% |
| EBV in situ hybridization |  |  |
| Positive | 0 | 0.0% |
| Negative | 25 | 54.3% |
| Unknown | 21 | 45.7% |
| MSI/MMR testing |  |  |
| MSI-H/dMMR | 1 | 2.2% |
| MSI-L/MSS/pMMR | 45 | 97.8% |
| PD-L1 status |  |  |
| CPS≥1 | 13 | 28.3% |
| CPS<1 | 13 | 28.3% |
| Unknown | 20 | 43.4% |

Abbreviations: ECOG, Eastern Cooperative Oncology Group; EGJ, esophagogastric junction; EBV, Epstein-Barr virus; MSI, microsatellite instability; MMR, mismatch repair; MSI-H, high-level microsatellite instability; MSI-L, low-level microsatellite instability; dMMR, deficient mismatch repair; pMMR, proficient mismatch repair; PD-L1,programmed cell death ligand 1.
