# Supplementary figures and images for: The predicting role of circulating tumor DNA landscape in gastric cancer patients treated with immune checkpoint inhibitors
Source: Mol Cancer. 2020 Oct 30;19:154. doi: 10.1186/s12943-020-01274-7 (PMC7596978; doi:10.1186/s12943-020-01274-7)

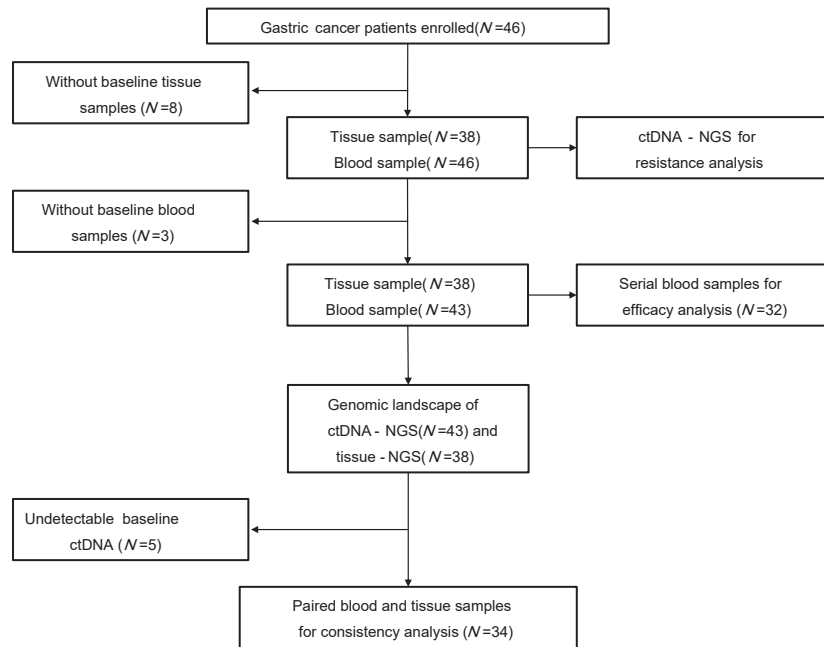

Supplement: Supplementary file 3 — Additional file 3: Figure S1. CONSORT diagram. CONSORT diagram of 46 patients enrolled and samples analyzed. [file 12943_2020_1274_MOESM3_ESM.pdf]

A

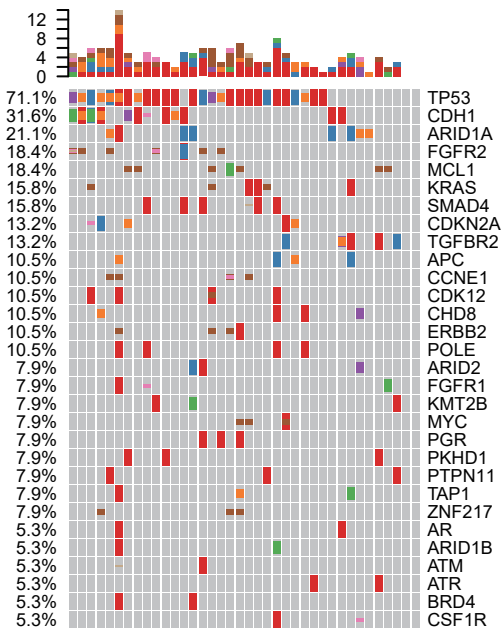

B

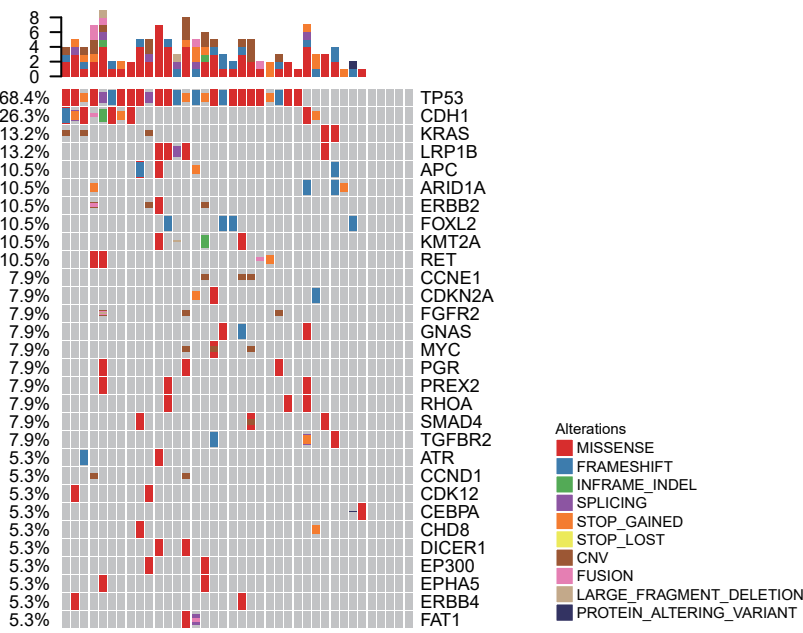

Supplement: Supplementary file 5 — Additional file 5: Figure S3. The landscape of high-frequency genomic alterations detected in 38 tissue samples and 38 baseline plasma samples. High-frequency means genomic alterations detected in tissue or baseline plasma > 5%. [file 12943_2020_1274_MOESM5_ESM.pdf]

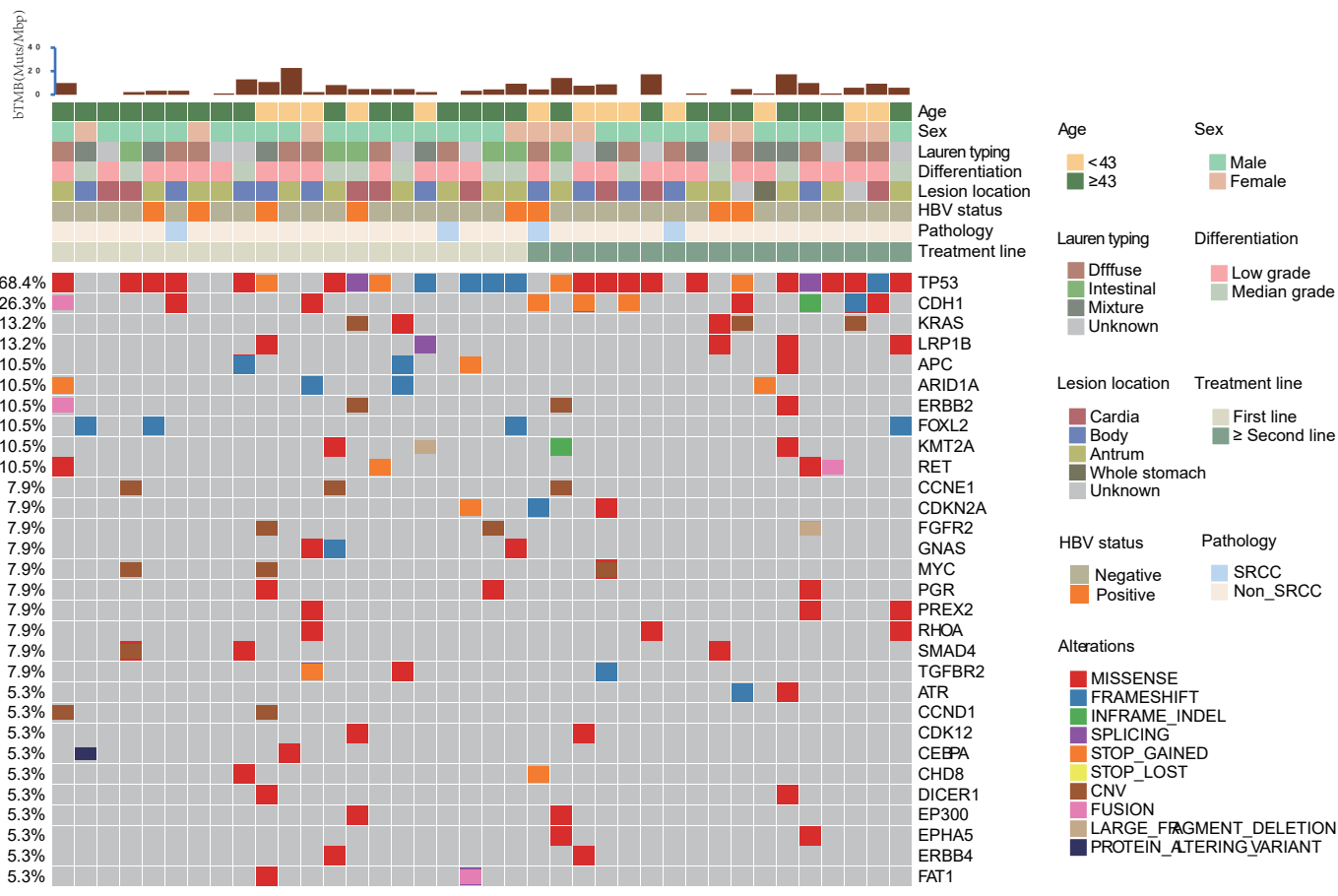

Supplement: Supplementary file 6 — Additional file 6: Figure S4. The characteristics of 38 patients with available baseline plasma samples. [file 12943_2020_1274_MOESM6_ESM.pdf]

A

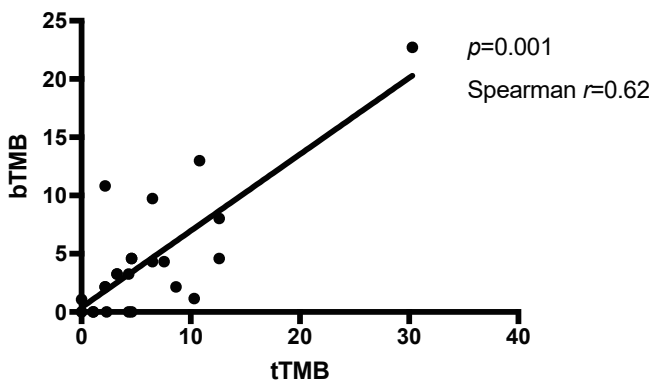

B

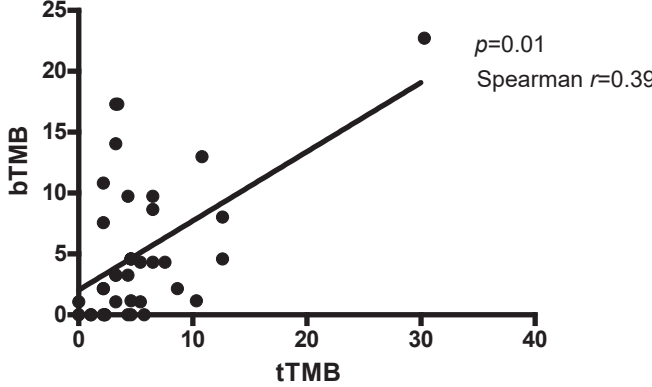

Supplement: Supplementary file 7 — Additional file 7: Figure S5. Scatter plot between tTMB and bTMB. The Spearman’s rank test showed significant correlations. (p < 0.05) a Scatter plot between tTMB and bTMB in 24 patients who received immunotherapy as first-line treatment. b Scatter plot between tTMB and bTMB in all 38 patients whose tissue and baseline plasma samples were available. [file 12943_2020_1274_MOESM7_ESM.pdf]
