# Supplementary material for: The predicting role of circulating tumor DNA landscape in gastric cancer patients treated with immune checkpoint inhibitors
Source: Mol Cancer. 2020 Oct 30;19:154. doi: 10.1186/s12943-020-01274-7 (PMC7596978; doi:10.1186/s12943-020-01274-7)
Supplement: Supplementary file 4 — Additional file 4: Figure S2. 425-gene NGS panel that used for plasma circulating tumor DNA sequencing and tissue tumor DNA sequencing. [file 12943_2020_1274_MOESM4_ESM.pdf]

ABCB1 (MDR1)	ABCB4	ADH1B	ADH1A	ABCC2 (MRP2)
ADH1C	AIP	AKT1	AKT2	AKT3
ALDH2	ALK	AMER1	APC	AR
ARAF	ARID1A	ARID1B	ARID2	ARID5B
ASCL4	ASXL1	ATF1	ATIC	ATM
ATR	ATRX	AURKA	AURKB	AXIN2
AXL	B2M	BAD	BAI3	BAK1
BAP1	BARD1	BAX	BCL2	BCL2L11 (BIM)
BCR	BIRC3	BLM	BMPR1A	BRAF
BRCA1	BRCA2	BRD4	BRIP1	BTG2
BTK	BUB1B	c11orf30	CASP8	CBL
CBLB	CCND1	CCNE1	CD74	CD274 (PD-L1)
CDA	CDC73	CDH1	CDK10	CDK12
CDK4	CDK6	CDK8	CDKN1A	CDKN1B
CDKN1C	CDKN2A	CDKN2B	CDKN2C	CEBPA
CEP57	CHD4	CHEK1	CHEK2	CREBBP
CRKL	CSF1R	CTCF	CTLA4	CTNNB1
CUL3	CUX1	CXCR4	CYLD	CYP19A1
CYP2A13	CYP2A6	CYP2A7	CYP2B6*6	CYP2C19*2
CYP2C9*3	CYP2D6	CYP3A4*4	CYP3A5	DAXX
DDR2	DENND1A	DHFR	DICER1	DLL3
DNMT3A	DPYD	DUSP2	EGFR	EP300
EPAS1	EPCAM	EPHA2	EPHA3	EPHA5
ERBB2 (HER2)	EPHB2	ERBB2IP	ERBB3	ERBB4
ERCC1	ERCC2	ERCC3	ERCC4	ERCC5
ESR1	ETV1	ETV4	ETV6	EWSR1
EXT1	EXT2	EZH2	FANCA	FANCC
FANCD2	FANCE	FANCF	FANCG	FANCI

FANCL	FANCM	FAT1	FBXW7	FGF19
FGFR1	FGFR2	FGFR3	FGFR4	FH
FLT1 (VEGFR1)	FLCN	FLT3	FLT4	FOXA1
FOXP1	FRG1	GATA1	GATA2	GATA3
GATA4	GATA6	GNA11	GNAQ	GNAS
GRIN2A	GRM3	GRM8	GSTM1	GSTM4
GSTM5	GSTP1	GSTT1	HDAC2	HDAC9
HGF	HLA-A	HNF1A	HNF1B	HRAS
HSD3B1	IDH1	IDH2	IFNG	IFNGR1
IGF1R	IGF2	IKBKE	IKZF1	IL7R
INPP4B	IRF2	JAK1	JAK2	JAK3
JARID2	JUN	KDM5A	KDM6A	KDR (VEGFR2)
KEAP1	KIF1B	KIF5B	KIT	KITLG
KMT2A (MLL)	KLLN	KMT2B	KMT2C	KMT2D (MLL2)
KRAS	LHCGR	LMO1	LRP1B	LYN
LZTR1	MAP2K4	MAP3K1	MAP3K4	MAP2K1 (MEK1)
MAP2K2 (MEK2)	MAP4K3	MAX	MCL1	MDM2
MDM4	MECOM	MED12	MEF2B	MEN1
MET	MGMT	MITF	MLH1	MLH3
MLLT1	MLLT3	MLLT4	MPL	MRE11A
MSH2	MSH6	MTHFR	MTOR	MUTYH
MYC	MYCL	MYCN	MYD88	MYH9
NAT1	NBN	NCOR1	NF1	NF2
NFE2L2	NFKBIA	NKX2-1	NKX2-4	NOTCH1
NOTCH2	NOTCH3	NPM1	NQO1	NRAS
NRG1	NSD1	NTRK1	NTRK2	NTRK3
PAK3	PALB2	PALLD	PARK2	PARP1
PARP2	PAX5	PBRM1	PDE11A	PDCD1 (PD1)
PDCD1LG2(PD-L2)	PDGFRA	PDGFRB	PDK1	PGR

PHOX2B	PIK3C3	PIK3CA	PIK3R1	PIK3R2
PKHD1	PLAG1	PLK1	PMS1	PMS2
POLD1	POLD3	POLE	POLH	POT1
PPARD	PPP2R1A	PRDM1	PRF1	PRKACA
PRKACG	PRKAR1A	PRKCI	PRKDC	PRSS1
PRSS3	PTCH1	PTEN	PTK2	PTPN11
PTPN13	PTPRD	QKI	RAC1	RAC3
RAD50	RAD51	RAD51B	RAD51C	RAD51D
RAD54L	RAF1	RARA	RARG	RASGEF1A
RB1	RECQL4	RELN	RET	RHOA
RICTOR	RNF43	ROS1	RPTOR	RRM1
RUNX1	RUNX1T1	SBDS	SDC4	SDHA
SDHB	SDHC	SDHD	SEPT9	SETBP1
SETD2	SF3B1	SGK1	SLC34A2	SLC3A2
SLC7A8	SMAD2	SMAD3	SMAD4	SMAD7
SMARCA4	SMARCB1	SMO	SOS1	SOX1
SOX14	SOX2	SOX21	SPOP	SPRY4
SRC	SRY	STAG2	STAT3	STK11
STMN1	STT3A	SUFU	TAP1	TAP2
TEK	TEKT4	TERC	TERT	TERT
TET2	TGFBR2	THADA	TMEM127	TMPRSS2
TNFAIP3	TNFRSF11A	TNFRSF14	TNFRSF19	TNFSF11
TOP1	TOP2A	TP53	TP63	TPMT
TSC1	TSC2	TSHR	TTF1	TUBB3
TUBB4A	TUBB4B	TUBB6	TYMS	U2AF1
UGT1A1	VAMP2	VEGFA	VHL	WAS
WISP3	WRN	WT1	XPA	XPC
XRCC1	YAP1	ZNF2	ZNF217	ZNF703
