# Supplementary material for: The predicting role of circulating tumor DNA landscape in gastric cancer patients treated with immune checkpoint inhibitors
Source: Mol Cancer. 2020 Oct 30;19:154. doi: 10.1186/s12943-020-01274-7 (PMC7596978; doi:10.1186/s12943-020-01274-7)
Supplement: Supplementary file 9 — Additional file 9: Table S2. The dynamic changes of ctDNA of patients had benefit from the treatment. [file 12943_2020_1274_MOESM9_ESM.docx]

**Table S2.** **The dynamic changes of ctDNA of patients had benefit from the treatment.**

| Patient ID | Response | MaxVAF of first plasma sample (%) | MaxVAF of second plasma sample (%) | MaxVAF of PD plasma sample (%) |
| --- | --- | --- | --- | --- |
| SYSUCC004 | SD | 8.1 | 0.1 | 4.6 |
| SYSUCC007 | PR | 76.9 | 3. 8 | 54.2 |
| SYSUCC008 | PR | 1.7 | 0 | 12.6 |
| SYSUCC012 | SD | 19.5 | 0 | 21.0 |
| SYSUCC017 | SD | 1.2 | 1.3 | 0.6 |
| SYSUCC018 | SD | 3.3 | 53.4 | 11.5 |
| SYSUCC019 | SD | 3.1 | 44.6 | 40. 8 |
| SYSUCC025 | SD | 5.4 | - | 45.7 |
| SYSUCC029 | SD | 5.5 | 1. 5 | 8.4 |
| SYSUCC030 | SD | 0 | - | 0.5 |
| SYSUCC031 | SD | 7.2 | 0.9 | 9.9 |
| SYSUCC032 | PR | 2.4 | - | 6.3 |
| SYSUCC041 | SD | 23.0 | 22.8 | 27.2 |
| SYSUCC042 | SD | 5.1 | 3.5 | 75.5 |
| SYSUCC044 | SD | 42.1 | 56.6 | 59.5 |
| SYSUCC045 | PR | 23.6 | 27.5 | 48.2 |

Abbreviations: MaxVAF, maximal somatic variant allelic frequency; PR, partial response; SD, stable disease; PD, progressive disease.
