# Supplementary material for: The predicting role of circulating tumor DNA landscape in gastric cancer patients treated with immune checkpoint inhibitors
Source: Mol Cancer. 2020 Oct 30;19:154. doi: 10.1186/s12943-020-01274-7 (PMC7596978; doi:10.1186/s12943-020-01274-7)
Supplement: Supplementary file 8 — Additional file 8: Figure S6. Pathological examination of the patient’s gastric carcinoma in PREX2 wild type and mutant patients. a,d Representative section of H.E. staining showing gastric carcinoma in wild type and mutant patients respectively. Scale bar: 100 μm. b,e The PREX2 expression in tumor and non-tumor regions in wild type and mutant patients respectively. Scale bar: 100 μm. c,f CD8+ T cell densities in wild type and mutant patients respectively. Scale bar: 100 μm. g Statistical analysis of PD-L1 expression in 25 MSS patients by combined positive score (CPS). h Comparisons of the number of CD8+ T cells in PREX2 wild type and mutant patients. Student’s t-test were used. p > 0.05 was not considered statistically significant. [file 12943_2020_1274_MOESM8_ESM.pdf]

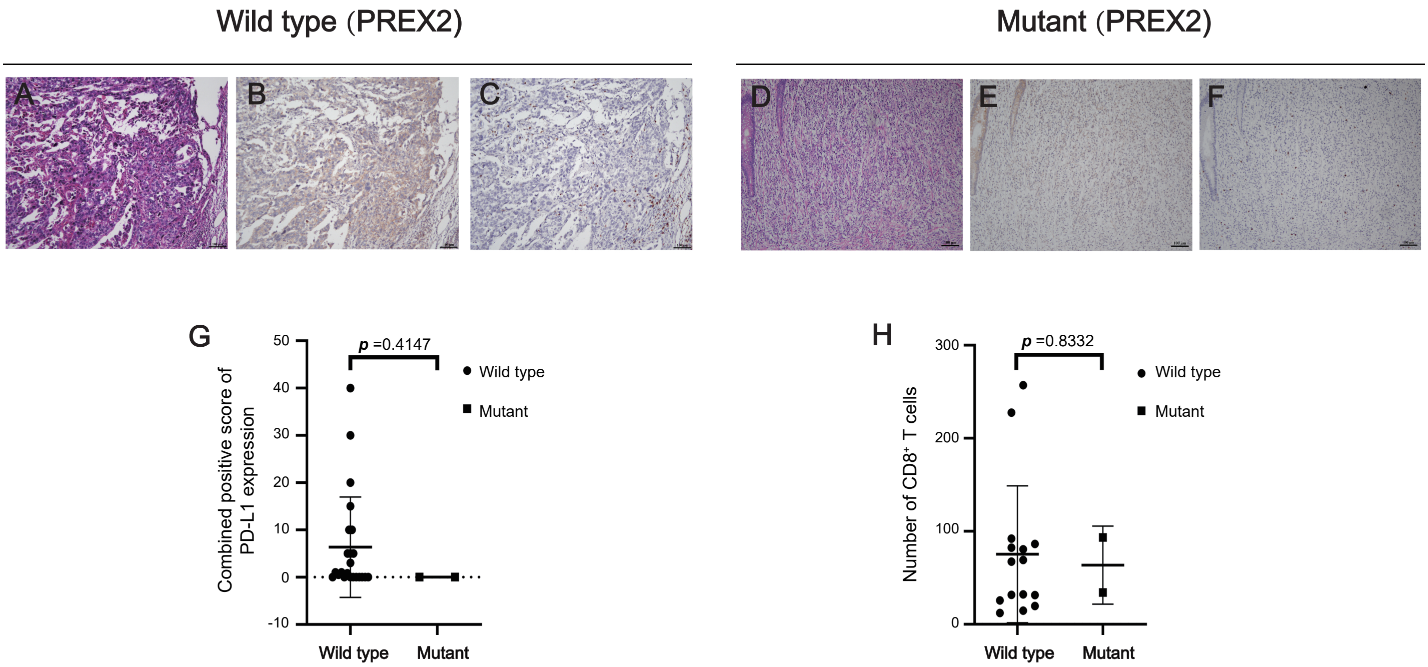

Wild type (PREX2)
Mutant (PREX2)
A
B
C
D
E
F
G
p =0.4147
Wild type
Mutant
Combined positive score of PD-L1 expression
50
40
30
20
10
0
-10
Wild type
Mutant
H
p =0.8332
Wild type
Mutant
Number of CD8+ T cells
300
200
100
0
Wild type
Mutant
